# Supplementary material for: Clock-Controlled Mitochondrial Dynamics Correlates with Cyclic Pregnenolone Synthesis
Source: Cells. 2020 Oct 19;9(10):2323. doi: 10.3390/cells9102323 (PMC7589815; doi:10.3390/cells9102323)
Supplement: Supplementary file 1 [file cells-09-02323-s001.pdf]

cells MDPI

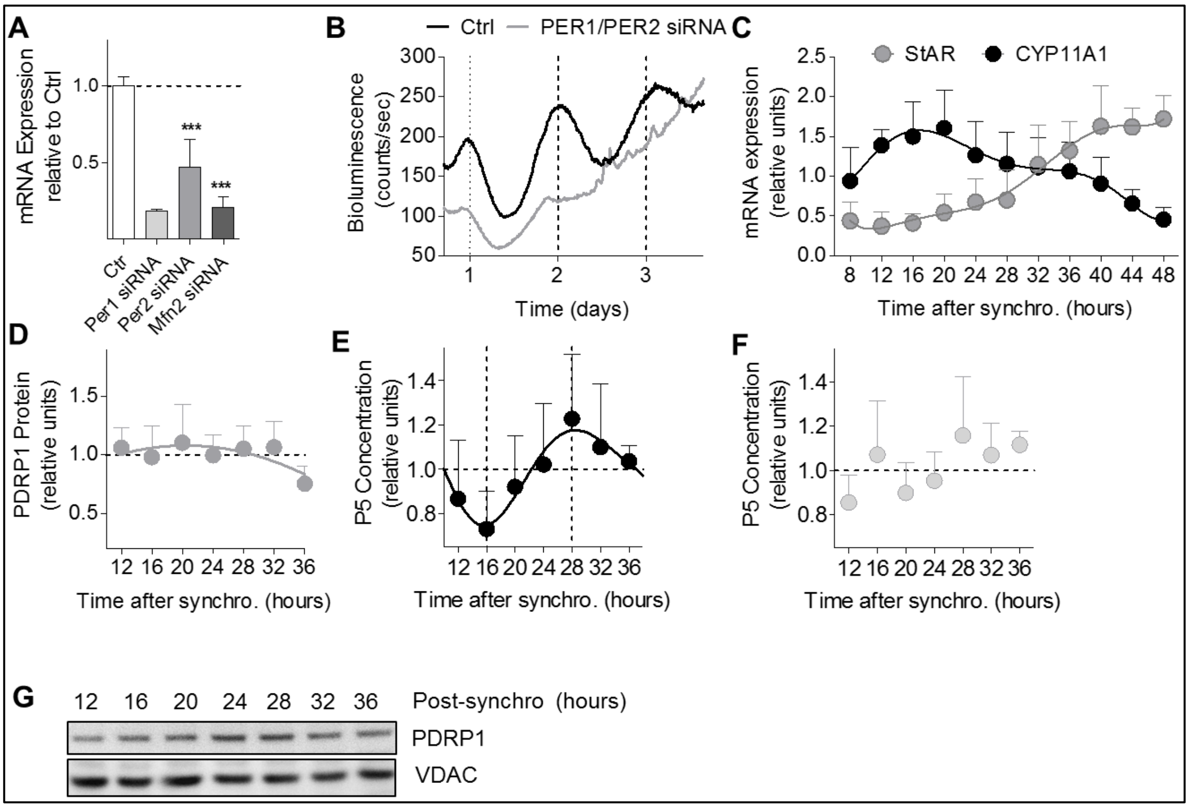

Supplementary Figure S1. Additional data related to Figure 1 and Figure 4. (**A**) PER1/PER2 siRNA transfection decreased PER1 and PER2 gene expression level (related to Figure 1). MFN2 siRNA transfection decrease MFN2 gene expression level (related to Figure 4). All data are normalized to negative Ctrl siRNA condition and an endogenous control and presented as mean ± SD (two experiment, *n* = 4). (**B**) PER1/PER2 siRNA transfection leads to arrhythmic BMAL1 gene expression compared to control. Representative bioluminescence of synchronized A172 glioma cells transfected with BMAL1::luciferase reporter in a LumiCycle. Cells are transfected with either PER1/PER2 siRNA or negative control siRNA (Ctrl). Raw data are plotted and presented as mean only (two experiment, *n* = 4 for each condition). (**C**) Relative StAR and CYP11A1 mRNA expression level quantified in synchronized A172 glioma cells using quantitative RT-PCR. Time points 8 to 48 h post-synchronization are shown. All data are normalized on a calibrator and an endogenous control and presented as mean ± SD (three independent experiments, seven time points, *n* = 11–12 for each time point). (**D**) Phospho-DRP1 was quantified in lysates of synchronized PER1/PER2 siRNA transfected A172 glioma cells. Time points from 12 to 36 h post-synchronization are indicated. All data are normalized to 1 and presented as mean ± SD (three experiment, seven time points, *n* = 4–6 for each time point). (**E**) P5 concentration was measured in lysates from synchronized Hela cells. Time points from 12 to 36 h post-synchronization are shown. All data are normalized to 1 and presented as mean ± SD (two independent experiments, seven time points, *n* = 4–6 for each time point). (**F**) P5 concentration was measured in lysates from synchronized Hela cells Drp1 knockout. Time points from 12 to 36 h post-synchronization are shown. All data are normalized to 1 and presented as mean ± SD (two independent experiments, seven time points, *n* = 4–6 for each time point). (**G**) Representative immunoblots of lysates from synchronized PER1/PER2 siRNA transfected cells stained with antibodies specific for phosphorylated DRP1 at serine 637 (PDRP1) and VDAC (control). Time points from 12 to 32 h post-synchronization are shown.

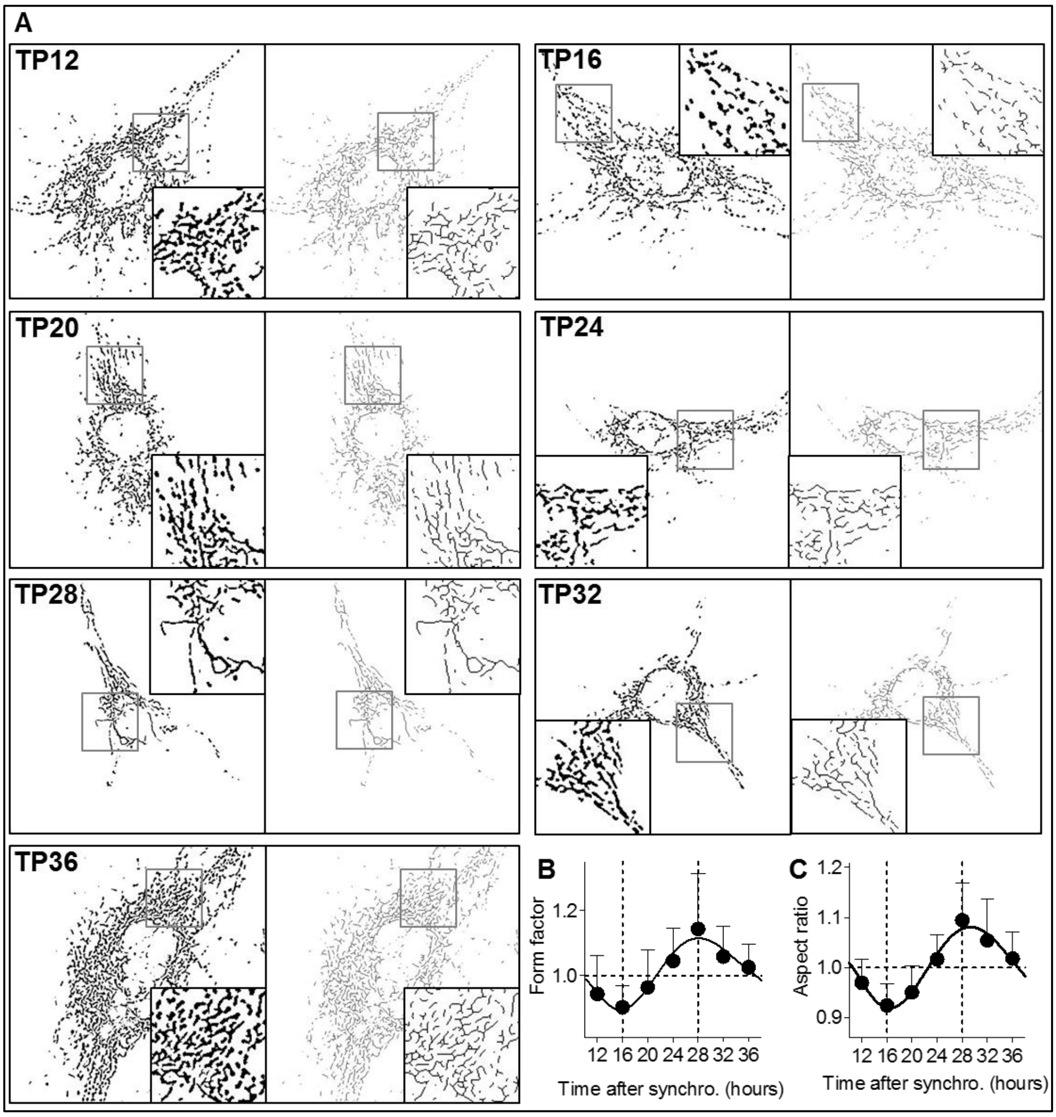

Supplementary Figure S2. Mitochondrial network morphology shows a circadian rhythmicity in A172 glioma cells (supplementary data). (**A**) Confocal images of rhythmically changing mitochondrial network morphology in synchronized A172 glioma cells transfected with mitochondrially-targeted GFP. Binary pictures (ImageJ "Otsu" threshold) and pictures obtained with the "Skeletonize" filter are shown. Representative images were acquired from 12 to 36 h post-synchronization. Time points TP12 and 16 correspond to a fragmented network, TP20 and 24 to an intermediate state and TP28 and 32 to a tubular, elongated network. Scale bars: 25 µm. (**B–C**) Quantification of mitochondrial "Form Factor" (**B**) and "Aspect Ratio" (**C**) (see details in the *Materials and Methods* part) in synchronized A172 glioma cells from 12 to 36 h post-synchronization. All data are normalized to 1 and presented as mean ± SD (two independent experiments, seven time points). About 40 images containing 3000–5000 mitochondria were analyzed per time point).

Supplementary Table S1: Summary of the analysis of circadian rhythmicity using the JTK_Cycle and One-way ANOVA (comparison of peak versus trough).

cells

MDPI

			JTK_Cycle p-value	Period length	Rhythmicity	One-way ANOVA + Tukey Peak versus Trough
Figure 1	A	**P5 Concentration**	**3.07E-08**	**24**	**CIRCADIAN**	*p* < 0.001 ***
	B	*P5 Concentration P1P2 KD*	*0.4643*		*NOT CIRCADIAN*	n.a.
	C	**TSPO protein**	**1.98E-06**	**24**	**CIRCADIAN**	*p* < 0.05 *
	D	*TSPO protein P1P2 KD*	*0.3191*		*NOT CIRCADIAN*	n.a.
Figure 2	A	**P5 Concentration Mice DD**	**5.56E-05**	**24**	**CIRCADIAN**	*p* < 0.01 **
	C	**TSPO protein Mice DD**	**0.000255146**	**24**	**CIRCADIAN**	*p* < 0.05 *
Figure 3	B	**Mito. Length**	**3.71E-07**	**24**	**CIRCADIAN**	*p* < 0.001 ***
	C	**PDRP1 protein**	**7.11E-07**	**24**	**CIRCADIAN**	*p* <0.05 *
Figure 5	A	*P5 Concentration Mdivi*	*0.53*		*NOT CIRCADIAN*	n.a.
	B	*P5 Concentration Mfn2 KD*	*0.22023682*		*NOT CIRCADIAN*	n.a.
	C	*TSPO Mdivi*	*0.823*		*NOT CIRCADIAN*	n.a.
	D	*TSPO Mfn2 KD*	*0.362728636*		*NOT CIRCADIAN*	n.a.
Supplementary Figure S1	C	CYP11A1 expression	0.065733008		*NOT CIRCADIAN*	n.a.
	C	StAR	0.298095		*NOT CIRCADIAN*	n.a.
	D	*PDRP1 P1P2 KD*	*0.8641.0560*		*NOT CIRCADIAN*	n.a.
	E	**P5 Concentration Hela**	**0.000779712**	**24**	**CIRCADIAN**	*p* < 0.05 *
	F	*P5 Concentration Hela Drp1 KO*	*0.7686996*		*NOT CIRCADIAN*	n.a.
Supplementary. Figure S2	B	**Form Factor**	**1.07E-08**	**24**	**CIRCADIAN**	*p* < 0.001 ***
	C	**Aspect Ratio**	**2.02E-16**	**24**	**CIRCADIAN**	*p* < 0.001 ***

In bold text: circadian rhythm, italic text: no circadian rhythm.

cells MDPI

Supplementary Materials and Methods S1 (related to Supplementary Figures). Material and Methods related to Supplementary Figure S1.

RNA isolation and quantitative real-time PCR

24 h prior to synchronization A172 glioma cells were seeded at a density of 0.08×10^6 cells/mL in DMEM + 1% penicillin/streptomycin + 1% GlutaMax + 10% FBS in 6-well plates (Falcon, #353046) and cells were harvested from 8 to 48 h post-synchronization every 4 h. Harvesting procedure and subsequent RNA isolation were performed using an RNeasy Mini Kit (QIAGEN, #74106) according to instructions of the manufacturer. Briefly, cells were harvested in 350 µL RLT lysis buffer after a washing step with PBS^-. Then, cell lysates were homogenized, 350 µL of 70% ethanol was added and samples were transferred to RNeasy spin columns. After several washing steps, RNA was eluted in 30 µL RNase-free water and isolated RNA amounts were determined using a Nanodrop 1000 spectrophotometer (Thermo Fisher Scientific). A calibrator was mixed containing equal amounts of samples from all time points. Next, RNA amounts were adjusted to 1 µg and reverse transcribed in cDNA using Ready-to-go you-prime first-strand beads (GE healthcare, #27926401) according to the instructions of the manufacturer. RNA was heated 10 min at 65°C, RNA amounts adjusted with DEPC-treated water (Ambion, Thermo Fisher Scientific, #AM9906) and 1 µL Oligo $(dT)_{15}$ primers (Promega, #C1101) were added (final volume of 33 µL). Samples were then incubated for 1 h at 37 °C. Resulting cDNAs were then used for quantitative real-time PCR, which was performed using a Step One Plus system (Applied Biosystems). Following primers were used: StAR (Taqman Gene Expression, Applied Biosystems, Thermo Fisher Scientific, # 4351370, assay ID #Hs00986559_g1), CYP11A1 (Taqman Gene Expression, Applied Biosystems, Thermo Fisher Scientific, # 4351370, assay ID # Hs00897320_m1) and Cdk4 (Microsynth, see Table Primer sequences). Cdk4 was used as endogenous control in data analysis. For data sets in supplementary figures following primers were used: PER1, PER2 (both Microsynth Table Primer sequences) and Mfn2 (Taqman Gene Expression, Applied Biosystems, Thermo Fisher Scientific, #4331182, assay ID #Hs00208382_m1). For quantitative real-time PCR, cDNA was diluted in nuclease-free water (Ambion, Thermo Fisher Scientific, #AM9937) 1:3 for TSPO, 1:2 for StAR, CYP11A1, PER1, PER2 and Mfn2. Then, a PCR mix was prepared consisting of DyNAmo Flash Probe master mix (DyNAmo Flash Probe qPCR kit, Thermo Fisher Scientific, #F-455XL), corresponding primers and nuclease-free water. Then, 3 µl diluted cDNA and 17 µl PCR mix were combined in each well and after a centrifugation step (10 s at 1000 rcf), quantitative real-time PCR was performed using the following parameters: initial 7 min at 95°C and then 45 cycles of 95°C for 5 s followed by 60°C for 30 s.

Primer sequences

Gene name	Primer sequences
Human PER1	Forward: 5′-CGCCTAACCCCGTATGTGA-3′<br>Reverse: 5′-CGCGTAGTGAAAATCCTCTTGTC-3′<br>Probe: 5′-Yakima Yellow-CGCATCCATTCGGGTTACGAAGCTC-BHQ1-3′
Human PER2	Forward: 5′-GGGCAGCCTTTCGACTATTCT-3′<br>Reverse: 5′-GCTGGTGTCCAACGTGATGTACT-3′<br>Probe: 5′-Yakima Yellow-CATTCGGTTTCGCGCCCGGG-BHQ1-3′
Human Cdk4	Forward: 5′-GAGGCGACTGGAGGCTTTT-3′<br>Reverse: 5′-GGATGTGGCACAGACGTCC-3′<br>Probe: 5′-FAM-AGC ATC CCA ATG TTG TCC GGC TGA-BHQ1-3′

Lumicycle Analysis

For period length determination, A172 glioma cells transfected with luciferase under control of the BMAL1 promoter were seeded at a density of 0.1 × 10^6 cells/mL in DMEM + 1% penicillin/streptomycin + 1% GlutaMax + 10% FBS in 3.5 cm dishes (Falcon, #353001) 24 h prior to synchronization. Cells were synchronized by addition of 100 nM dexamethasone for 15 min at 37 °C [38]. Then, cells were washed three times with PBS and placed in DMEM without phenol red (Sigma, Sigma-Aldrich, #D1145) + 1% penicillin/streptomycin, 1% GlutaMax, 5% FBS, 10 mM HEPES, 1% sodium pyruvate (Sigma-Aldrich, #S8636), 4.5 g/L glucose (Sigma-Aldrich, #D6134) and 0.1 mM luciferin (Thermo Fisher Scientific, #L2912). For at least 5 days, the amount of produced light that is proportional to BMAL1 gene expression was measured using a LumiCycle 32 instrument (Actimetrics). Data were analysed with Lumicycle Analysis software (LumicycleTM, version 2.31, Actimetrics Software) and the period of oscillation was calculated by least-mean-squares fitting of dampened sine wave functions to the actual data as described previously [39].
